# From algorithm to verification: based on network toxicology and machine learning, the immunomodulatory role of IGFBP1/MKI67/C9 in perfluorooctanoic acid-induced osteoarthritis was discovered, and a diagnostic model was constructed

**DOI:** 10.3389/fimmu.2026.1700638

**Published:** 2026-05-28

**Authors:** Xinzhou Huang, Yongkun Wei, Yani Rao, Yue Wei, Hui Chen, Yunping Bao

**Affiliations:** 1 Department of Orthopedics, 3201 Hospital of Xi’an Jiaotong University Health Science Center, Hanzhong, China; 2 The First Clinical Medical College of Yangtze University, Jingzhou, China; 3 Department of Laboratory, The First People’s Hospital of Jingzhou (First Affiliated Hospital of Yangtze University), Jingzhou, China; 4 Department of Orthopedics, The First People’s Hospital of Jingzhou (First Affiliated Hospital of Yangtze University), Jingzhou, China

**Keywords:** bioinformatics analysis, immune infiltration, machine learning, molecular docking, osteoarthritis, perfluorooctanoic acid, WGCNA

## Abstract

**Background:**

Perfluorooctanoic acid (PFOA), a widespread persistent environmental contaminant, has been associated with osteoarthritis (OA) onset and progression, though mechanisms remain unclear. This study elucidates PFOA’s influence on OA pathogenesis, evaluates its effects on disease progression, and identifies diagnostic biomarkers.

**Method:**

Obtain PFOA and OA-related gene expression data from public databases, integrate GSE114007 and GSE89408, and perform batch correction. Differentially expressed genes were identified via limma for GO and KEGG enrichment. Six machine learning algorithms (Lasso, SVM, Boruta, XGBoost, LightGBM, AdaBoost) and WGCNA screened key genes. Expression of candidate genes in OA synovial tissue was verified by qRT-PCR, and a diagnostic nomogram was constructed and evaluated. Immune cell infiltration was analyzed by ssGSEA, and molecular docking studied PFOA binding to target proteins.

**Result:**

15 PFOA-related OA differentially expressed genes were identified. Machine learning and WGCNA determined IGFBP1, MKI67 and C9 as core genes; qRT-PCR verified they were significantly upregulated in OA patients. Enrichment analysis revealed involvement in inflammatory, immune and metabolic processes. Immune infiltration analysis indicated multiple immune cells significantly increased in OA samples; core genes helped inhibit excessive Th17 and B cell responses while enhancing Treg and NKT regulatory activity. Molecular docking showed strong binding of PFOA to the three core proteins (binding energies: -6.0, -8.5, -7.5 kcal/mol). The nomogram achieved AUC of 0.903 in training set and 0.939 in external validation set (GSE51588).

**Conclusion:**

PFOA exposure may be associated with OA immune microenvironment alterations, potentially involving dysregulation of IGFBP1, MKI67 and C9, contributing to inflammation and cartilage degradation. These three genes are promising diagnostic biomarkers for OA, providing new insights into environmental pollutant involvement in OA pathogenesis.

## 1 Introduction

Perfluorooctanoic acid (PFOA) is a synthetic perfluorinated compound that has garnered significant attention due to its pronounced bioaccumulation and persistent environmental presence (1). Industrial production of PFOA commenced in the 1940s, and it has been utilized extensively across various commercial and industrial domains, including non-stick coatings, high-performance polymer processing, food contact packaging, and the manufacture of fire retardant foams (2). The environmental prevalence of PFOA primarily originates from its extensive application in industrial processes and the consequent large-scale emissions, coupled with the widespread use of consumer products that contain these compounds as impurities. These factors collectively contribute to its substantial accumulation in natural ecosystems (3). Furthermore, the presence of exceptionally strong carbon-fluorine bonds in PFOA, recognized as the most robust among all covalent bonds, imparts considerable stability against various degradation processes. This inherent stability facilitates its pervasive accumulation and bioaccumulation within the environment (4).

Numerous studies have investigated the health implications of PFOA through *in vivo* research. Accumulation of PFOA in animals has been associated with multisystemic toxicity, encompassing genetic, immunological, neurological, and hepatic effects (5–8). For example, developmental and reproductive toxicities have been reported in animal models (9, 10). These findings suggest that PFOA can disrupt normal cellular proliferation and metabolic regulation, processes also implicated in joint tissue homeostasis.

Regarding hepatotoxicity, PFOA exposure has been shown to induce liver damage through PPAR-α pathway-mediated oxidative stress (11). Of note, PPAR-α signaling and oxidative stress are also key contributors to cartilage degradation and synovial inflammation in osteoarthritis (OA). Regarding malignancy, PFOA exposure has been associated with testicular and pancreatic tumors in rats, and can promote breast cancer cell invasiveness via ERα/GPER signaling (12). These observations indicate that PFOA can aberrantly activate proliferation-related pathways (e.g., PI3K/AKT, MAPK/ERK), which are also implicated in synovial hyperplasia and chondrocyte dysfunction in OA.

Notably, recent cross-sectional studies have identified a positive association between elevated serum PFOA levels and OA incidence, particularly in females (13, 14). While PFOA toxicity has been extensively studied in other organs, experimental studies on its role in OA remain scarce, and the precise mechanisms are unclear. OA, a prevalent chronic joint disorder primarily impacting weight-bearing joints like the knees and hips, is characterized by the progressive degeneration of articular cartilage, bone remodeling, synovial inflammation, and the formation of bone spurs, often resulting in pain and functional impairment (15). According to recent data from the World Health Organization, OA impacts over 500 million individuals globally, with its incidence and prevalence increasing in correlation with aging and escalating risk factors (16). The condition predominantly affects the elderly and women (17). Contributing factors to OA include aging, gender, obesity, genetic predisposition, trauma, joint deformities, and environmental influences (18, 19). Proactively identifying the detrimental effects of PFOA on OA could enhance the optimization of treatment strategies for patients. However, there are only a few widely recognised biomarkers for OA prediction.

This study conducts a thorough bioinformatics analysis of the influence of PFOA on OA progression, utilizing multiple online databases to investigate its mechanism of action, associated genes, and complex biological pathways, with the objective of assessing its potential impact on human health. Through the application of Gene Ontology (GO) and Kyoto Encyclopedia of Genes and Genomes (KEGG) pathway enrichment analyses, the study elucidates the potential biological pathways and disease implications linked to PFOA. Furthermore, an efficacious diagnostic model was developed to assess the impact of PFOA on genes associated with OA. A practical nomogram was also proposed to facilitate potential clinical applications. Preliminary experimental validation was conducted to substantiate these findings.

This study aims to clarify how PFOA affects OA progression by disrupting the gene networks of IGFBP1, MKI67, and C9. Understanding these interactions is crucial for identifying the impact of environmental pollutants on joint diseases. IGFBP1, a key factor in insulin-like growth factor signaling, may be involved in OA pathogenesis, and its expression changes may correlate with PFOA exposure, suggesting a potential link to metabolic disturbances and extracellular matrix degradation in chondrocytes. Research shows that PFOA exposure can disrupt IGFBP1-regulated glycolipid metabolism, worsen abnormal bone remodeling, and increase synovial inflammation. The exact impact of PFOA on apoptosis and autophagy imbalance in OA chondrocytes is still unclear. MKI67 is a key marker of cell proliferation and has been found to be overexpressed in samples of osteoarthritis. The increase in its expression level may reflect the excessive activation of fibroblasts or the abnormal proliferation of chondrocytes, which may be related to PFOA exposure and the accelerated degeneration of joints. Transcriptome analysis shows PFOA increases MKI67 expression by activating oxidative stress pathways like NF-κB. Further research is needed to understand its link to cartilage calcification or osteophyte formation in OA. C9 is a key molecule in the complement pathway and has been found to exhibit differential expression in OA patients. Its abnormal activation may be related to PFOA/PFOS exposure and may contribute to chronic inflammation. This can lead to synovial macrophage polarization and release of pro-inflammatory cytokines like IL-1β and TNF-α, resulting in cartilage damage. Network toxicology suggests PFOA might influence C9 expression through DNA methylation, but its role in altering the OA-specific immune environment needs further validation.

This study uses multi-omics analysis and *in vitro* models to explore how the PFOA-IGFBP1/MKI67/C9 axis affects OA pathology. It reveals molecular links between pollutants and joint diseases, supporting the development of targeted therapies for metabolic, cell cycle, and immune regulation. The research into IGFBP1’s role in cartilage metabolism, MKI67 in joint repair, and C9 in inflammation could offer new insights into chronic diseases caused by environmental toxins. This study uses network toxicology, transcriptome maps, and functional modules to explore how PFOA causes OA through gene networks. This analysis improves understanding of environment-gene interactions and aids early detection and targeted intervention for OA.

## 2 Methods

### 2.1 PFOA-associated genes screening and online databases investigation

The Comparative Toxicogenomics Database (CTD) serves as an integrative platform that consolidates diverse datasets encompassing chemical substances, genes, functional phenotypes, and disease associations. This repository has significantly advanced the investigation of the relationships between environmental exposures and diseases, as well as the exploration of potential drug mechanisms of action, thereby substantially contributing to scientific progress in related disciplines. From the CTD, all genes interacting with PFOA were extracted. Subsequently, a total of 379 PFOA-related genes were identified from the CTD database, based on a relevance score exceeding 4. Two OA gene expression microarray datasets (GSE114007 and GSE89408) from NCBI Gene Expression Omnibus (GEO; https://www.ncbi.nlm.nih.gov/geo/). The GSE114007 dataset utilizes the GPL11154 platform, Illumina HiSeq 2000 (Homo sapiens), and the GPL18573 platform, Illumina NextSeq 500 (Homo sapiens), comprising 18 normal samples and 20 OA samples. In contrast, the GSE89408 dataset is based on the GPL11154 platform, Illumina HiSeq 2000 (Homo sapiens), and includes 28 normal samples and 22 OA samples. Utilizing R (version 4.3.1), the ‘sva’ package was employed to eliminate batch effects and integrate the GSE114007 and GSE89408 datasets (20). Subsequently, Principal Component Analysis was conducted to evaluate the efficacy of batch effect removal in the GSE114007 and GSE89408 datasets and to visualize the distribution of OA and normal patient samples. It should be noted that the GEO datasets (GSE114007 and GSE89408) used in this study did not contain information on PFOA exposure history of the participants. Therefore, our analysis can only identify molecular associations between PFOA−related genes and OA, rather than establishing a direct causal relationship between PFOA exposure and OA pathogenesis. We conducted an independent external validation by obtaining the GSE51588 dataset from the GEO database (platform: GPL13497, consisting of 10 normal samples and 40 samples of OA).

### 2.2 Identification of differentially expressed genes (DEGs) and related enrichment analysis

Differentially expressed genes (DEGs) between OA patients and control subjects were identified by analyzing the combined datasets (GSE114007 and GSE89408) utilizing the ‘limma’ package in R (21). The selection criteria for DEGs included a *p*-value of less than 0.05 and an absolute log2 fold change (FC) > 1. Visualization of DEGs in OA was achieved through the generation of heatmaps and volcano plots, employing the ‘heatmap’ and ‘ggplot2’ packages, respectively. Subsequently, DEGs associated with PFOA, termed DEPFOAs, were identified by intersecting the DEGs with PFOA-related genes. We conducted functional and pathway analyses of OA-related DEGs utilizing the clusterProfiler R package, which encompassed GO and KEGG analyses (22). In addition, we performed GO and KEGG analyses of DEPFOAs using the enrichr online website (https://maayanlab.cloud/Enrichr/).

### 2.3 Machine learning

To achieve a more comprehensive identification and screening of key diagnostic factors, an array of advanced machine learning algorithms is employed for in-depth analysis. Specifically, a combination of Lasso regression, support vector machine (SVM), the Boruta algorithm, XGBoost, LightGBM, and AdaBoost is utilized. Lasso regression, in particular, effectively reduces model complexity through the introduction of L1 regularization, thereby filtering out features that significantly impact prediction outcomes. This algorithm is implemented using the R package ‘glmnet’ (23). SVMs, in contrast, classify data by utilizing hyperplanes that maximize the margin between different classes, and they are adept at addressing nonlinear problems within high-dimensional feature spaces. Conversely, Boruta’s algorithm is a feature selection method grounded in random forests, which assesses the importance of all features comprehensively rather than focusing on the localized perspective of an individual decision tree. This approach ensures that the features selected possess global significance. XGBoost and LightGBM are two highly efficient gradient boosting decision tree algorithms that inherently filter out features significantly impacting the model’s predictive efficacy. In contrast, AdaBoost is a boosting algorithm based on weak classifiers, which iteratively modifies the weights of the training data to ensure that subsequent models concentrate more on samples misclassified by previous models, thereby progressively enhancing the model’s overall performance. These algorithms are implemented through machine learning functions available in the R package ‘caret’ (24). Utilize ‘feature importance’ as a screening criterion to identify key biomarkers from genetic data. Subsequently, conduct an analysis of the genes that are common to six machine learning models, with particular emphasis on those genes that demonstrate superior performance across multiple algorithms.

### 2.4 WGCNA obtained feature module genes

Gene co-expression networks were developed and analyzed utilizing the R package ‘WGCNA’ to identify gene expression characteristics that exhibit strong correlations with OA (25). Initially, genes demonstrating stable expression levels were selected, while those with low median absolute deviation, comprising approximately 50% of the total, were excluded. To ensure the robustness of the network construction, the soft threshold power (β) was determined using the pickSoftThreshold function, ensuring an exponential fit greater than 0.9. The network was established by first calculating the adjacency matrix among genes, which was then transformed into a topological overlap matrix to improve the interpretability of the network connections. Hierarchical clustering was conducted utilizing the standard R function, hclust, to organize gene modules into branches of a clustered dendrogram. To further refine the analysis, the cutreeDynamic function was applied to identify and delineate the independent gene modules. Subsequent to this process, the mergeCloseModules function was employed to amalgamate the highly correlated modules, culminating in the identification of 17 distinct gene modules. To investigate the biological significance of these modules, the characteristic genes of the 17 modules underwent correlation analysis with OA, with the objective of elucidating their clinical relevance. Pearson correlation analysis was utilized to examine the associations between the gene modules and the clinical features of OA, resulting in the identification of gene modules that exhibit significant associations with OA. Subsequently, intersections of these core modules were identified with high-value genes selected through machine learning algorithms to accurately determine key genes for the study. Furthermore, the Kruskal-Wallis non-parametric test was employed to evaluate potential differences in continuous variables between the OA and control groups.

### 2.5 Patients samples

In this study, synovial tissue samples were obtained from six patients diagnosed with osteoarthritis during total knee arthroplasty, as well as from six patients with meniscus injuries during knee arthroscopy procedures. All participants provided voluntary informed consent, having been fully briefed on the study, with the consent process approved by the Ethics Committee of 3201 Hospital in Hanzhong City, China. We acknowledge that the sample size for qRT−PCR validation (n=6 per group) is relatively small, which may limit the representativeness of the findings. This experiment was designed as a preliminary validation to support the bioinformatics results, rather than as a definitive clinical validation.

### 2.6 qRT-PCR and gene expression analysis

To conduct a comprehensive investigation into the differences in gene expression between OA and normal knee synovial tissues, total RNA was isolated and extracted from both osteoarthritic and normal knee synovial tissues using the TRIzol reagent (Invitrogen, California, USA) according to a standardized protocol. The RNA purity was further improved through a purification process involving isopropanol precipitation, washing with 75% ethanol, and solubilization in RNase-free water. Following the accurate assessment of RNA concentration and purity, reverse transcription was conducted utilizing the Takara PrimeScript^®^ RT kit to synthesize complementary DNA (cDNA). The cDNA was then analyzed through quantitative real-time polymerase chain reaction (qRT-PCR) employing the 2× SYBR Green qPCR Master Mix (low ROX). The GAPDH gene served as an internal reference to normalize the data and mitigate inter-sample variability. To improve the reliability and validity of the experimental results, each biological sample was analyzed in triplicate technical replicates. The sequences of IGFBP1 were as follows: TTGGGACGCCATCAGTACCTA (sense) and TTGGCTAAACTCTCTACGACTCT (antisense). The sequences of MKI67 were as follows: GCTCACGCCTGTAATCCC (sense) and TGCTCTTCGCTTTGCTTT (antisense). The sequences of C9 were as follows: CGACTTCGGTGTAATGGTGAC (sense) and ATAGCCTGCTGTTCGTGCC (antisense). The sequences of GAPDH were as follows: TCCACCACCCTGTTGCTGTA (sense) and GACTTCAACAGCAACTCCCAC (antisense).

### 2.7 Construction and validation of a nomogram model and GSEA enrichment analysis

In the domain of OA diagnostics, column-line diagrams serve as an effective instrument for predictive modeling due to their intuitive characteristics. Utilizing the rms package in the R programming language, a prediction model was successfully developed, incorporating distinct genetic markers (26). Each genetic marker was quantitatively assessed to represent its specific contribution to OA status prediction, and the composite score synthesized the predictive capacity of each gene. To thoroughly assess the model, decision curve analysis (DCA) was employed to ascertain its decision-making value, while calibration curves were utilized to evaluate the concordance between the model’s predictions and the actual outcomes, thereby ensuring the model’s reliability and stability. The discriminative power of key genes was quantified through the construction of a receiver operating characteristic (ROC) curve, with the area under the curve (AUC) serving to validate the model’s sensitivity and specificity. Furthermore, gene set enrichment analysis (GSEA) was conducted on the core set of genes using the ‘clusterProfiler’ software package in R (27).

### 2.8 Immunological characteristics of OA

To investigate the pathological features of OA patients, this study employed single-sample Gene Set Enrichment Analysis (ssGSEA) to precisely evaluate the infiltration status of 28 immune cell types in OA patients compared to healthy controls (28). Visualization was achieved through the use of box line plots generated with the R language vioplot package. Additionally, the intrinsic relationship between the expression levels of key genes and the degree of immune cell infiltration was examined using Spearman rank correlation analysis.

### 2.9 Molecular docking

The interactions between specific genes and small molecule compounds were systematically evaluated utilizing advanced molecular docking analysis (29). To achieve this objective, the crystal structures of the target proteins IGFBP1, MKI67, and C9 were obtained from the Protein Data Bank, while the molecular structure of PFOA was sourced from the PubChem database. Both the receptor proteins and ligand small molecules underwent pre-processing with AutoDockTools software, which involved the precise addition of hydrogen atoms and the accurate positioning of active sites. Three-dimensional visualization was conducted utilizing PyMOL software. Following this, AutoDock Vina software was employed to elucidate the interaction patterns between the candidate proteins and the small molecule compounds, thereby identifying the critical sites and conformations involved in compound binding. The docking score served as the evaluation parameter for the molecular docking results, where lower values signified a higher affinity of the ligand for a specific protein target and greater stability of the resulting complex.

### 2.10 Statistical analysis

The algorithm was implemented using version 4.4.1 of the R programming language. Statistical differences between the two groups of continuous variables were analyzed using Student’s t-test, facilitated by GraphPad Prism version 7.0 software. A p-value of less than 0.05 was considered indicative of statistical significance.

## 3 Results

### 3.1 Identification and enrichment analysis of DEGs in OA

**Figure 1:**
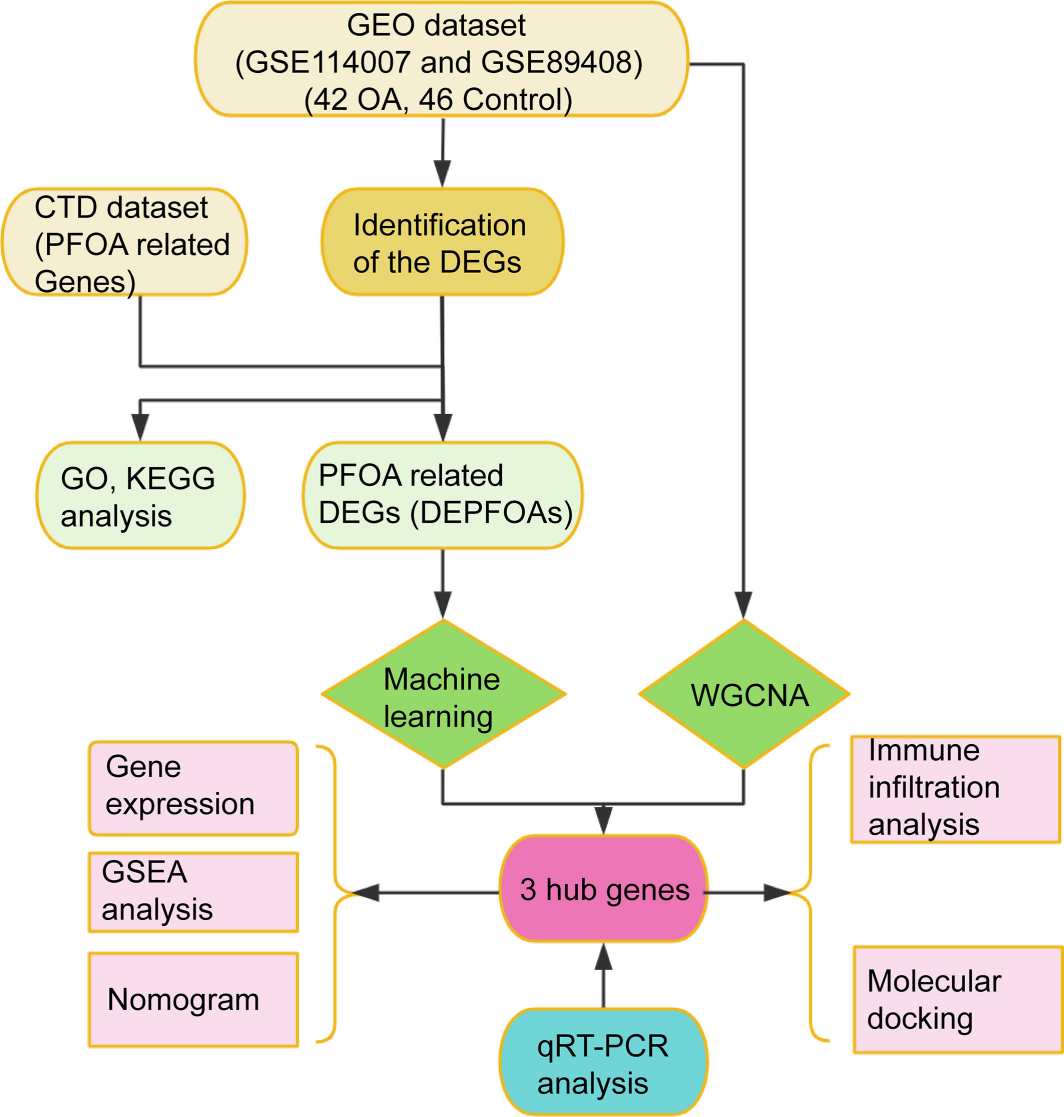
Flow-chart of datasets analysis in this paper.

**Figure 2:**
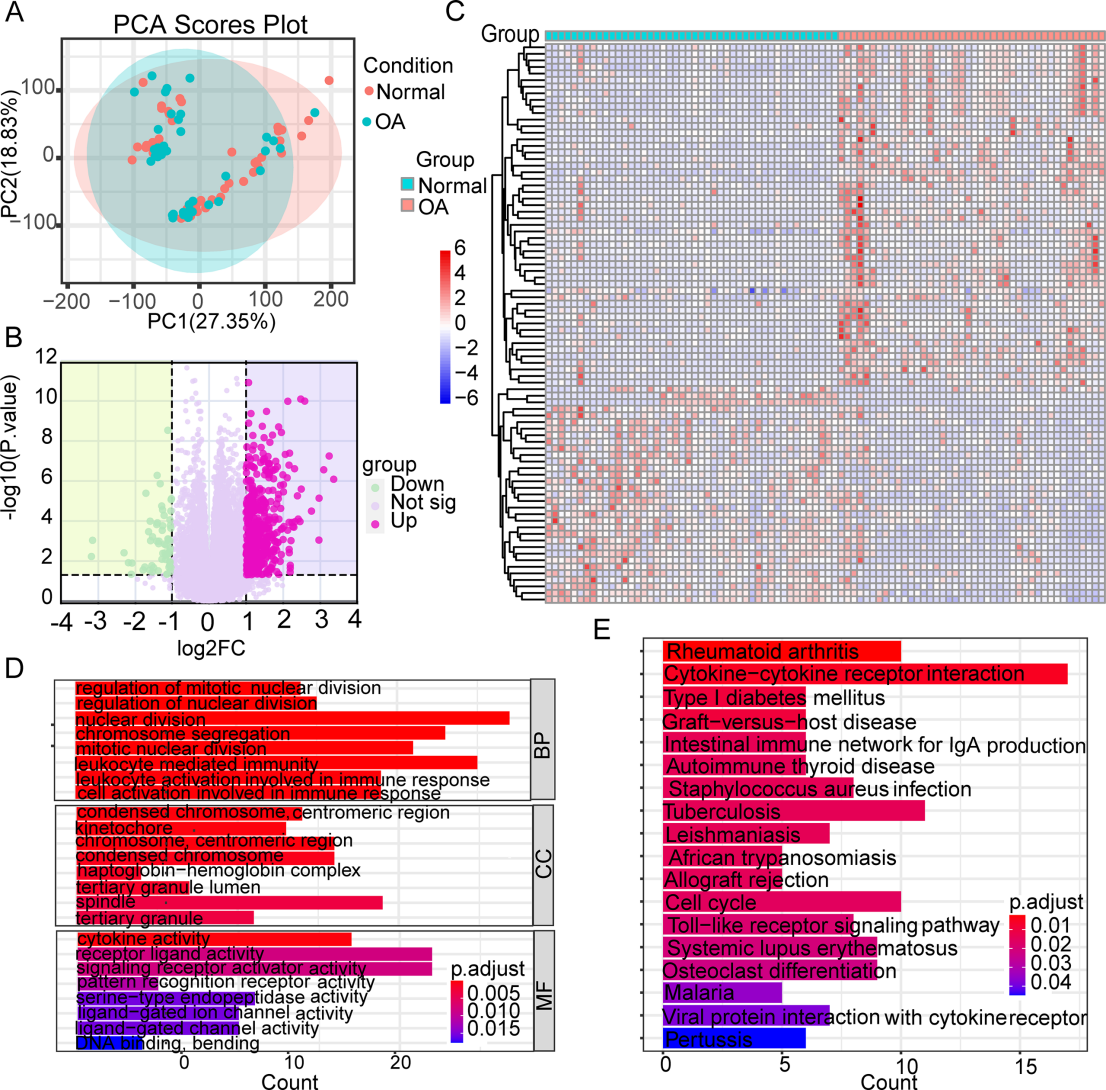
Differential gene expression and functional enrichment analysis between samples. **(A)** The principal component analysis (PCA) displaying a distinct profile between OA and control groups in GSE114007 and GSE89408. **(B)** Volcano plot of differentially expressed genes by comparing in GSE114007 and GSE89408. The red color indicates up-regulated genes, green indicates down-regulated genes, and purple indicates genes with similar expression between both groups. **(C)** Clustering analysis and heatmap of the DEGs between OA and control groups. **(D)** The bar chart representing the GO enrichment of DEGs, including biological process (BP), cellular component (CC), and molecular function (MF). **(E)** The bar chart showing the KEGG enrichment analysis of DEGs.

To elucidate the bioinformatics analysis methodology employed in this study, a summary is provided in **Figure 1**. To construct a comprehensive overview of genes associated with OA across all samples, we amalgamated the expression profiles from two datasets, GSE114007 and GSE89408, into a single, unified dataset. Recognizing that datasets from disparate sources frequently exhibit significant batch effects, we utilized R version 4.3.2 for the analysis of the raw data, subsequently applying batch effect correction and log normalization procedures. Following the successful mitigation of batch effects via principal component analysis, the dataset’s samples exhibited consistent and acceptable homogeneity (**Figure 2A**). This dataset comprised 42 OA samples and 46 control samples. Subsequent differential expression analysis provided a comprehensive overview of gene expression in OA, revealing that 336 genes were up-regulated and 35 genes were down-regulated (**Supplementary Table 1**). Two types of visualizations, namely volcano plots and heat maps, were employed to characterize the expression profiles of DEGs in OA (**Figures 2B, C**). Subsequently, GO and KEGG enrichment analyses were conducted to elucidate the biological functions associated with these DEGs and the underlying signaling pathways. GO enrichment analysis revealed that among 311 differentially expressed genes, the most significantly enriched biological processes included nuclear division (GO:0000280, 27/311 genes, 8.7%, *p* = 3.16×10^-8^, FDR = 3.8×10^-5^), chromosome segregation (GO:0007059, 23/311 genes, 7.4%, *p* = 9.86×10^-8^, FDR = 8.0×10^-5^), regulation of mitotic nuclear division (GO:0007088, 14/311 genes, 4.5%, *p* = 8.93×10^-9^, FDR = 3.1×10^-5^), and leukocyte-mediated immunity (GO:0002443, 25/311 genes, 8.0%, *p* = 2.18×10^-7^, FDR = 1.3×10^-4^). Regarding cellular components, DEGs were significantly enriched in condensed chromosome, centromeric region (GO:0000779, 14/322 genes, 4.3%, *p* = 9.90×10^-7^, FDR = 3.4×10^-4^), kinetochore (GO:0000776, 13/322 genes, 4.0%, *p* = 2.86×10^-6^, FDR = 4.0×10^-4^), chromosome, centromeric region (GO:0000775, 16/322 genes, 5.0%, *p* = 3.49×10^-6^, FDR = 4.0×10^-4^), and condensed chromosome (GO:0000793, 16/322 genes, 5.0%, *p* = 1.07×10^-5^, FDR = 9.2×10^-4^). For molecular functions, DEGs were significantly enriched in cytokine activity (GO:0005125, 17/312 genes, 5.4%, *p* = 6.38×10^-7^, FDR = 3.5×10^-4^), receptor ligand activity (GO:0048018, 22/312 genes, 7.1%, *p* = 3.52×10^-5^, FDR = 8.1×10^-^³), and signaling receptor activator activity (GO:0030546, 22/312 genes, 7.1%, *p* = 4.35×10^-5^, FDR = 8.1×10^-^³) (**Figure 2D**; **Supplementary Table 2 Sheet 1**). KEGG enrichment analysis demonstrated that DEGs were significantly enriched in Rheumatoid arthritis (hsa05323, 10/167 genes, 6.0%, *p* = 1.0×10^-5^, FDR = 2.4×10^-^³), Cytokine-cytokine receptor interaction (hsa04060, 17/167 genes, 10.2%, *p* = 4.5×10^-5^, FDR = 5.3×10^-^³), Type I diabetes mellitus (hsa04940, 6/167 genes, 3.6%, *p* = 1.4×10^-4^, FDR = 9.4×10^-^³), and Graft-versus-host disease (hsa05332, 6/167 genes, 3.6%, *p* = 1.6×10^-4^, FDR = 9.4×10^-^³) (**Figure 2E**; **Supplementary Table 2 Sheet2**).

### 3.2 Identification and enrichment analysis of PFOA related DEGs (DEPFOAs)

**Figure 3:**
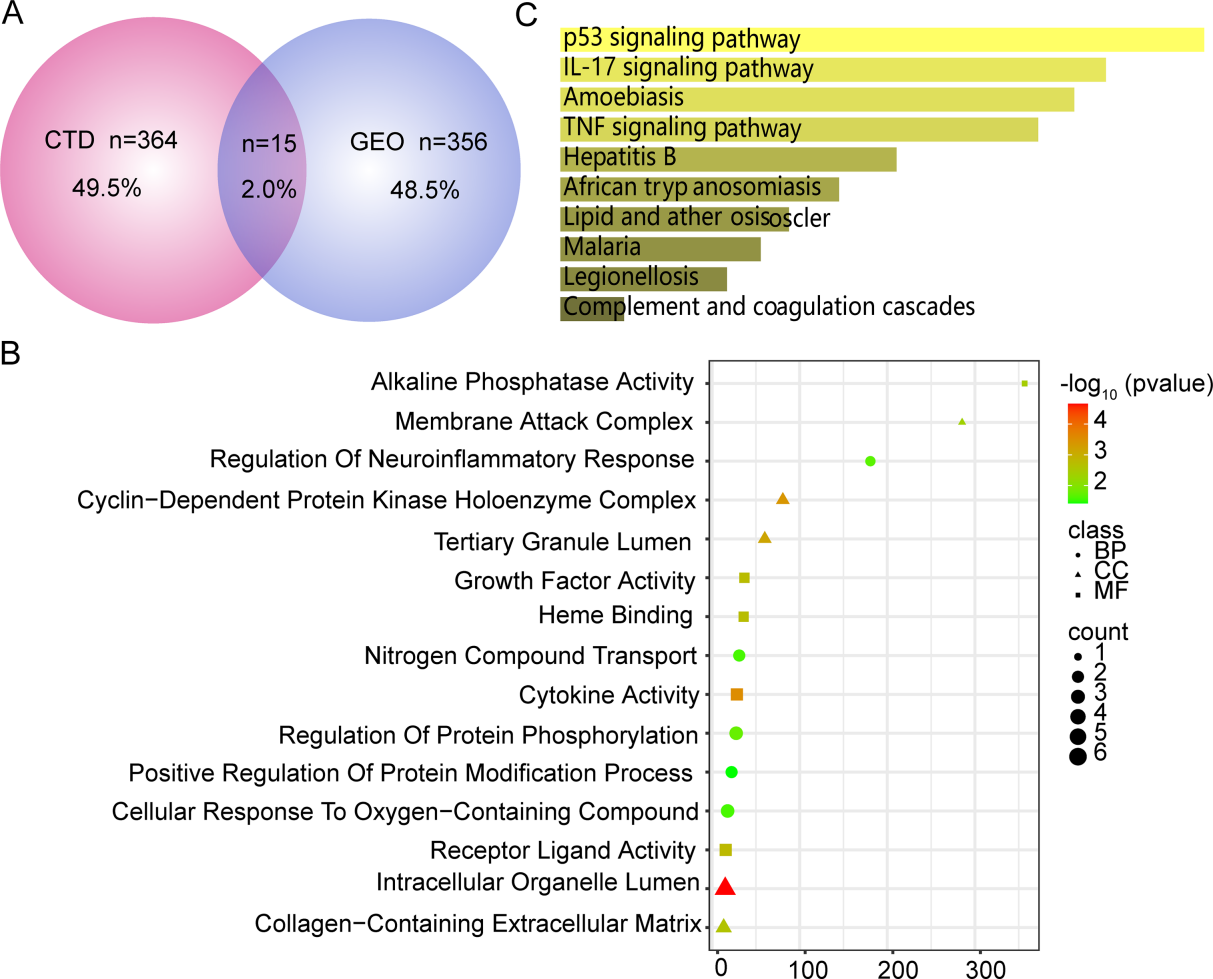
Identification and functional enrichment analyses of DEPFOAs. **(A)** The shared genes between the DEGs and PFOAs were represented in the Venn diagram. **(B)** GO enrichment analyses. **(C)** KEGG analysis annotation. The Venn diagram, bubble chart, and heatmap provide a comprehensive view of the potential biological functions and pathways associated with the genes at the intersection of DEGs and PFOAs, offering insights into their roles in OA pathogenesis.

Following the intersection of 371 differentially expressed genes associated with OA and 379 genes related to PFOA, a total of 15 DEPFOAs were identified (**Figure 3A**). These genes include AQP9, BIRC5, CCNE1, IGFBP1, CDK1, RRM2, F7, CXCL1, MKI67, IL1B, NRG1, ALPL, C9, MMP9, and HBA1. As illustrated in **Supplementary Figure S1**, significant correlations among the DEPFOAs were determined using Spearman correlation analysis. GO analysis indicated that the DEPFOAs are involved in processes such as “Regulation of Neuroinflammatory Response,” “Nitrogen Compound Transport,” and “Regulation of Protein Phosphorylation,” which were significantly enriched in the biological process category (**Figure 3B**). The KEGG analysis revealed that DEPFOAs are enriched in the p53 signaling pathway, IL-17 signaling pathway, TNF signaling pathway, and the lipid and atherosclerosis pathway (**Figure 3C**). Consequently, investigating the mechanisms of PFOA is crucial for advancing our understanding of the pathological progression of OA and for identifying potential therapeutic strategies.

### 3.3 Identification of diagnostic markers for OA

**Figure 4:**
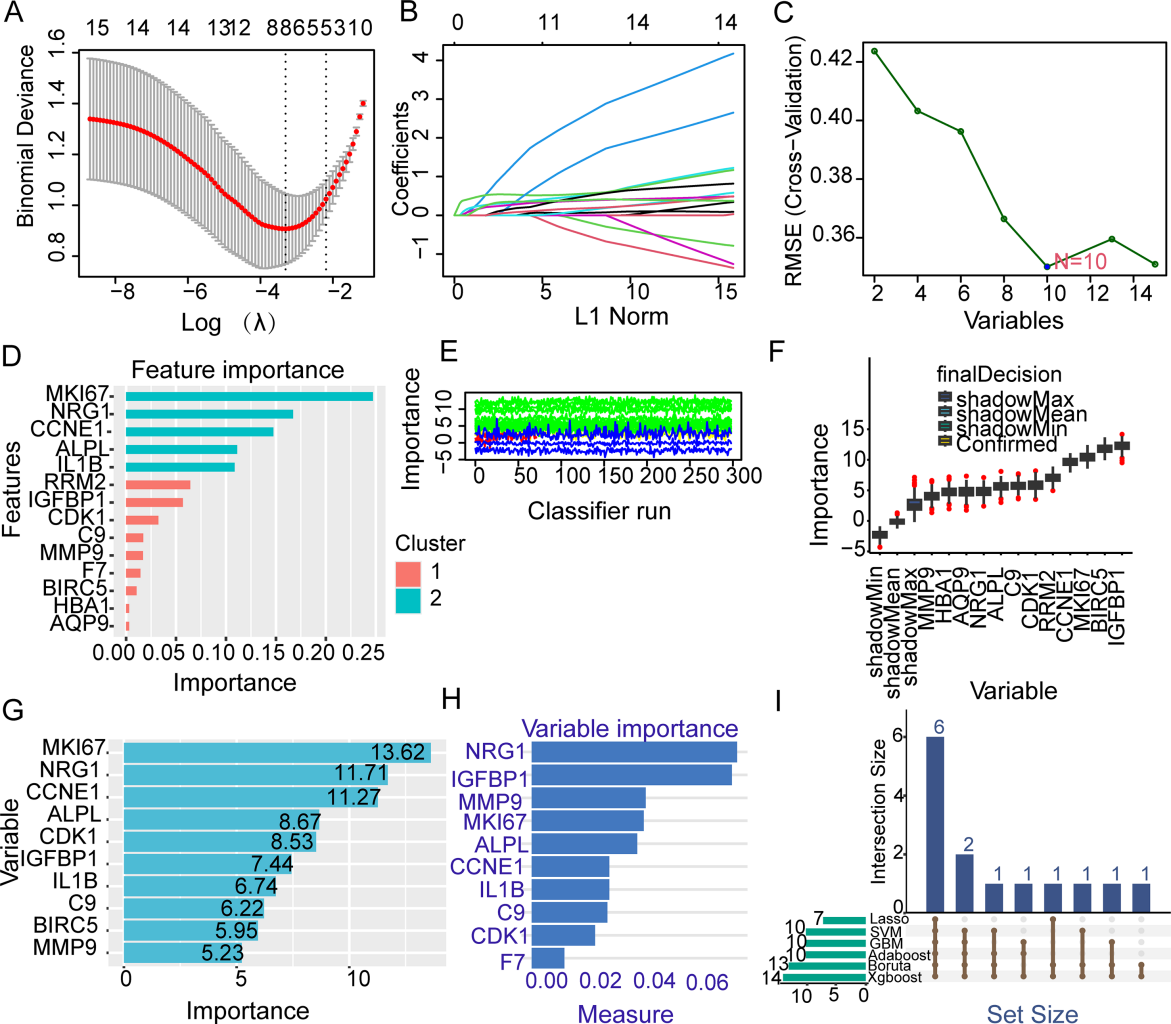
Screening of relevant diagnostic markers by six machine learning algorithms. **(A)** Lasso coefficient profiles of the seven diagnostic markers. **(B) **The partial likelihood deviation curve of the minimum number of diagnostic markers. **(C)** Screening of the ten diagnostic markers by SVM-RFE algorithm. **(D)** Screening of the fourteen diagnostic markers by XGBoost algorithm. **(E)** Evolution of the attribute Z-score during the Boruta run. **(F)** Thirteen diagnostic markers screened by the Boruta algorithm. **(G)** Ten diagnostic markers screened by the AdaBoost algorithm. **(H)** Ten diagnostic markers screened by the LightGBM algorithm. **(I)** Upset plots of the six machine learning intersections.

Six machine learning techniques were employed to identify biomarkers associated with OA induced by PFOA, utilizing hub genes as input variables. The findings indicated that the Lasso method successfully identified seven biomarkers, whereas the Support Vector Machine method identified ten biomarkers (**Figures 4A–C**). To assess the significance of these hub genes, additional machine learning methods were applied, including XGBoost (**Figure 4D**), Boruta (**Figures 4E, F**), AdaBoost (**Figure 4G**), and LightGBM (**Figure 4H**). Boruta identified the initial 13 genes as diagnostic markers for OA, while AdaBoost highlighted 10 genes with an importance score exceeding 5, and LightGBM also identified 10 genes. Through a comprehensive analysis of the outputs from six machine learning models, we identified six pivotal genes, namely IGFBP1, CDK1, MKI67, ALPL, C9, and MMP9 (**Figure 4I**).

### 3.4 WGCNA network construction and module gene screening

**Figure 5:**
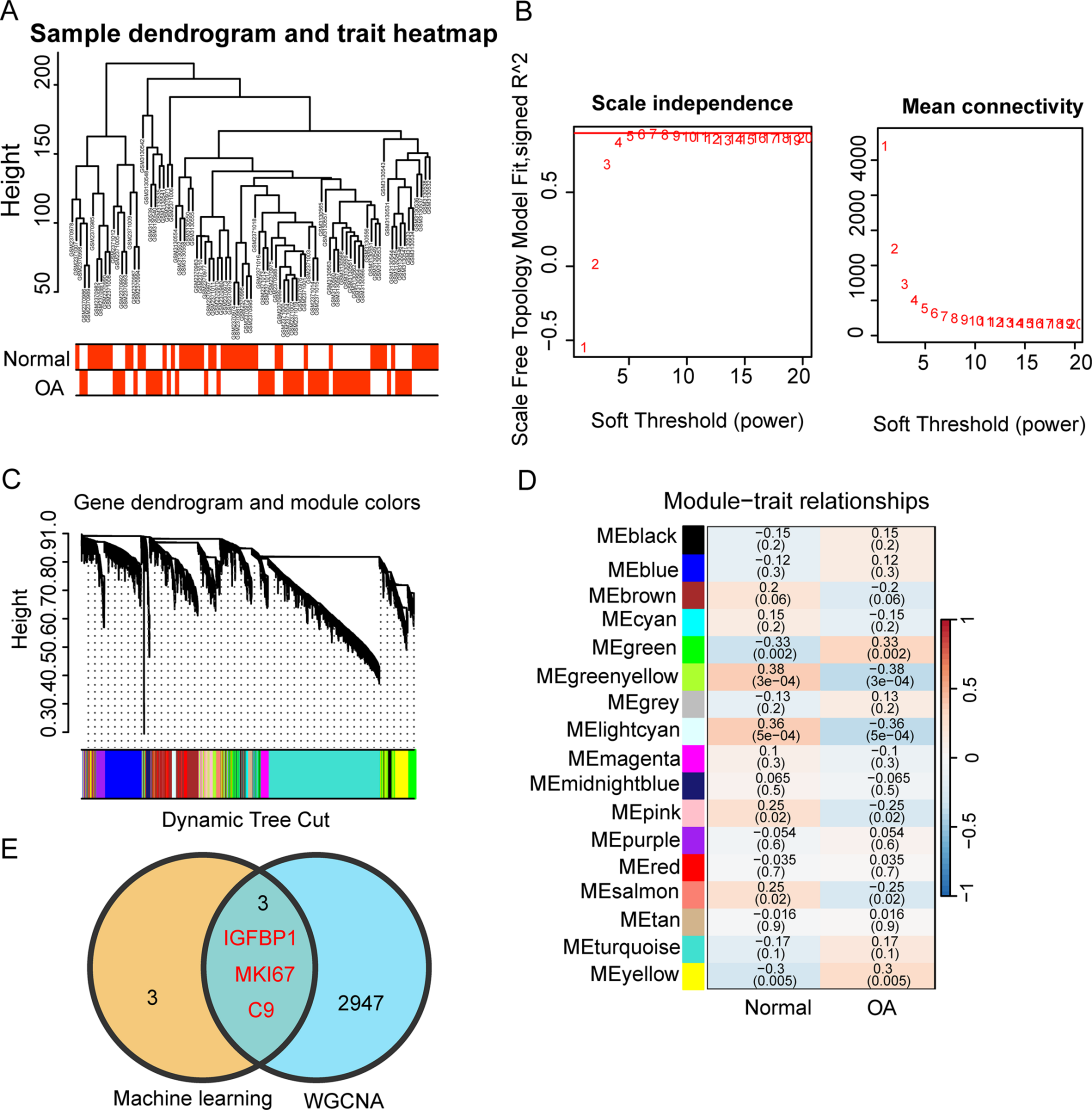
Module analysis of weighted gene co-expression network analysis (WGCNA). **(A)** Sample clustering to exclude outlier samples. **(B)** Network topology analysis under various soft-threshold powers. Left: The soft-threshold power. Right: The soft-threshold power. **(C)** Dendrogram clustering of gene modules according to 1-TOM value. **(D)** Heatmap of correlation between module characteristic genes and sample characteristics. The red table shows a positive correlation, and the blue indicates a negative correlation. **(E)** Venn diagram of the intersection between the genes selected by the machine learning module and the significant module genes of WGCNA.

Utilizing WGCNA to examine the dataset, 17 gene modules were identified that exhibited significant associations with OA. The sample clustering dendrogram, encompassing 46 normal specimens and 42 OA specimens, along with their corresponding clinical characteristics, is depicted through heat maps (**Figure 5A**). A power value of 5 was selected as the optimal soft threshold (r² = 0.873) for constructing a scale-free network (**Figure 5B**). Furthermore, module-trait association analyses were performed to pinpoint specific modules correlated with OA (**Figures 5C, D**). The green module, comprising 1,388 genes (r = 0.33, *p* = 0.002), and the yellow module, consisting of 1,562 genes (r = 0.3, *p* = 0.005), exhibited the strongest positive correlations with OA. Furthermore, an intersection of the genes identified by the gene module and the machine learning module revealed three common genes: IGFBP1, MKI67, and C9, as illustrated in **Figure 5E**.

### 3.5 qRT-PCR validation of hub gene

**Figure 6:**
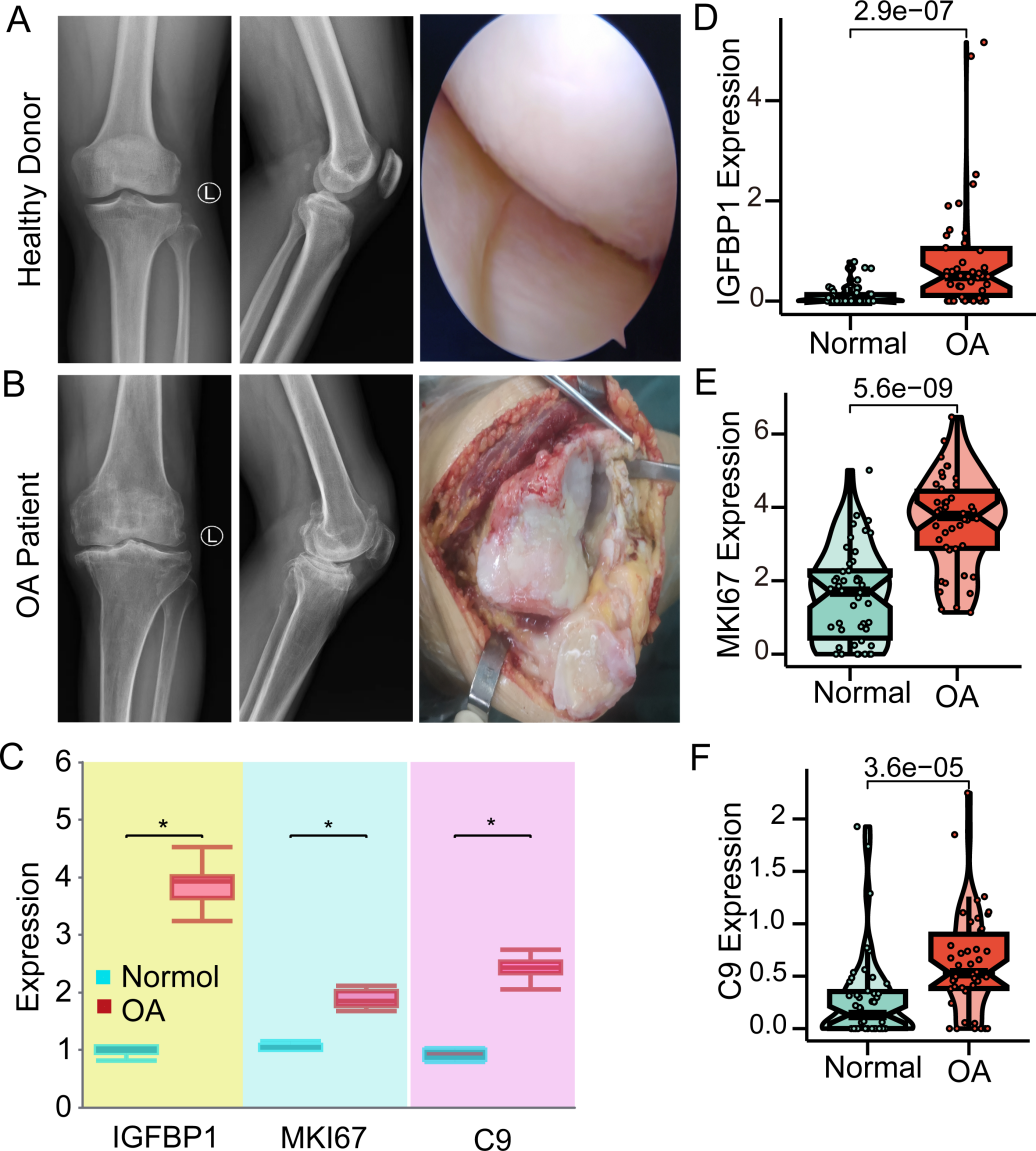
qRT-PCR validation of key Gene. **(A, B)** X ray images and intraoperative macroscopic views (arthroscopy image and arthroplasty) of knee joint from OA patients and healthy donors. **(C)** Validation of IGFBP1, MKI67 and C9 mRNA expression by qRT-PCR between OA (n = 6) and normal group (n = 6). **(D–F)** The expression comparison of three hub genes (IGFBP1, MKI67 and C9) in the GSE114007 and GSE89408 dataset. *p < 0.05.

The knee joints of each patient cohort underwent professional radiological evaluation to identify characteristic imaging features of OA across different patient groups. Through meticulous imaging analysis and macroscopic observation during surgical procedures, it was determined that the knee joints of OA patients exhibit significant pathological alterations compared to those of healthy individuals. Preoperative radiographic assessments of patients with OA revealed notable joint space narrowing, subchondral bone dysplasia, increased bone density, and structural sclerosis. Intraoperatively, extensive synovial hyperplasia, substantial osteophyte formation, degeneration of articular cartilage, surface roughening, loss of luster, and even detachment of cartilage fragments were visually observed (**Figures 6A, B**). The qRT-PCR results indicated that, in comparison to the normal group, the mRNA expression levels of IGFBP1, MKI67, and C9 were significantly up-regulated, aligning with the expression patterns observed in the training set (**Figure 6C**). Analysis of key gene expression levels within the combined dataset revealed a significant upregulation of IGFBP1, MKI67, and C9 in all osteoarthritis samples (**Figures 6D–F**).

### 3.6 Construction of the diagnostic nomogram for OA

**Figure 7:**
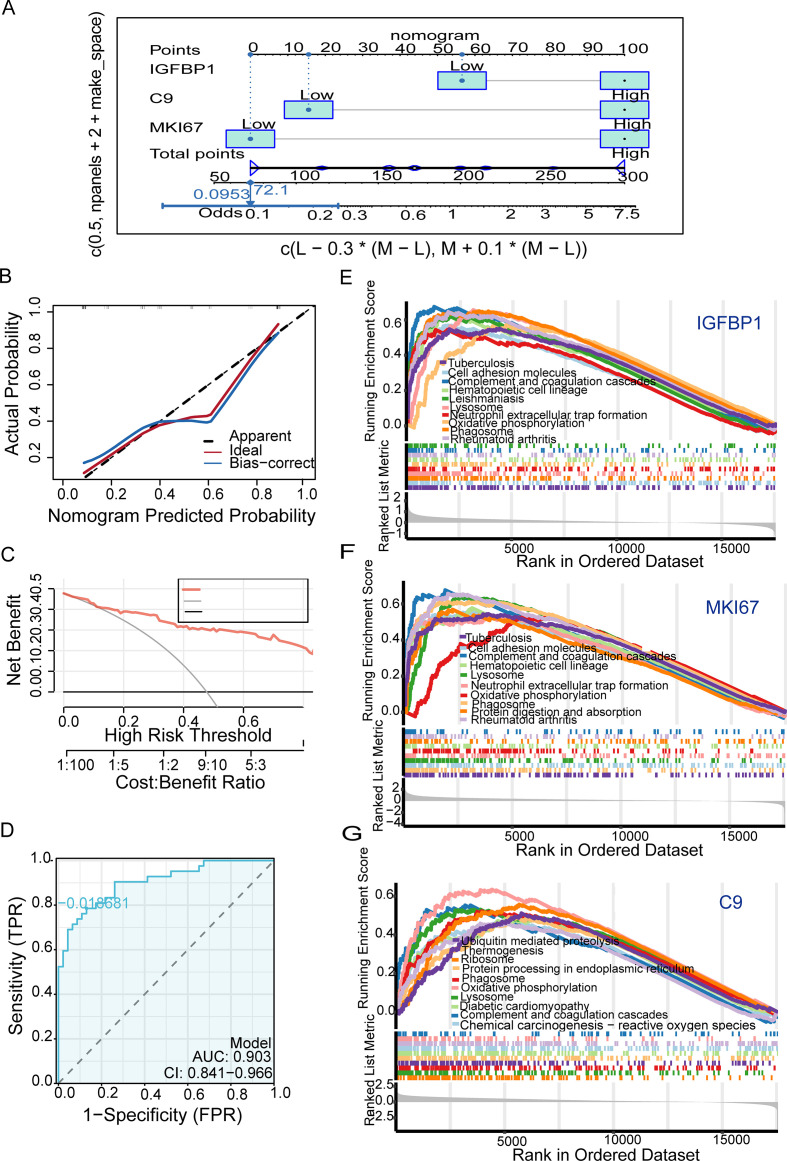
Construction of the diagnostic nomogram and diagnostic performance assessment and GSEA. **(A)** The diagnostic nomogram based on three characteristic genes was constructed. Each gene corresponded to a score, and the total score of the three genes was used to predict the risk of dilated OA. **(B)** The calibration curve was established to evaluate the accuracy of the nomogram. **(C)** The decision curve analysis was carried out to evaluate the net benefit of diagnostic decision of OA predicted by the nomogram. **(D)** Receiver operating characteristic curve (ROC) curves showing the performance of nomogram in predicting OA. **(E)** GSEA enrichment analysis of IGFBP1. **(F)** GSEA enrichment analysis of MKI67. **(G)** GSEA enrichment analysis of C9.

Utilizing the “rms” package, we developed an OA diagnostic model centered on characteristic genes. In the construction of the nomogram prediction model, each gene marker was allocated a distinct score weight, accurately representing its contribution to OA risk prediction. The scores of the three pivotal genes were aggregated to compute the total score, effectively illustrating the probability of OA incidence in individuals (**Figure 7A**). The construction of the correction curve and the application of the established nomogram model to predict the resultant probability distribution demonstrate a high level of consistency and alignment with the expected probabilities of the theoretical ideal model (**Figure 7B**). Furthermore, the results of the DCA clearly illustrate that clinical decision-making informed by our nomogram model significantly enhances the effectiveness of decision-making compared to traditional predictive models or the absence of predictive measures (**Figure 7C**). The nomogram, founded on these three key genes, demonstrated superior diagnostic performance, achieving an AUC value of 0.903 (**Figure 7D**). Independent validation in GSE51588 yielded an AUC of 0.939 (95% CI: 0.846–1.000) (**Supplementary Figure S2**).

### 3.7 Single-gene GSEA of characteristic genes

To enhance our understanding of the biological functions and signal transduction pathways associated with IGFBP1, MKI67, and C9, we conducted a single-gene GSEA to examine the functional roles of each gene individually. Single-gene GSEA revealed that IGFBP1 was significantly enriched in cell adhesion molecules (NES = 0.56, *P*-adj < 0.001), complement and coagulation cascades (NES = 0.66, *P*-adj < 0.001), and neutrophil extracellular trap formation (NES = 0.53, *P*-adj < 0.001). Similarly, MKI67 showed significant enrichment in cell adhesion molecules (NES = 0.57, *P*-adj < 0.001) and rheumatoid arthritis pathways (NES = 0.67, *P*-adj < 0.001) (**Figures 7E, F**). Notably, these pathways are known to be influenced by PFOA exposure, which can activate complement cascades and promote neutrophil extracellular trap formation through oxidative stress mechanisms. C9 was enriched in oxidative phosphorylation (NES = 0.63, *P*-adj < 0.001) and complement cascades (NES = 0.55, *P*-adj < 0.001) (**Figure 7G**). The enrichment of reactive oxygen species-related pathways is particularly relevant, as PFOA has been reported to induce oxidative stress, potentially linking environmental exposure to complement activation and metabolic dysregulation in OA. Results from single-gene GSEA indicate that central genes may play a significant role in the regulation of immune infiltration, inflammation, and metabolic processes.

### 3.8 Immune infiltration analysis

**Figure 8:**
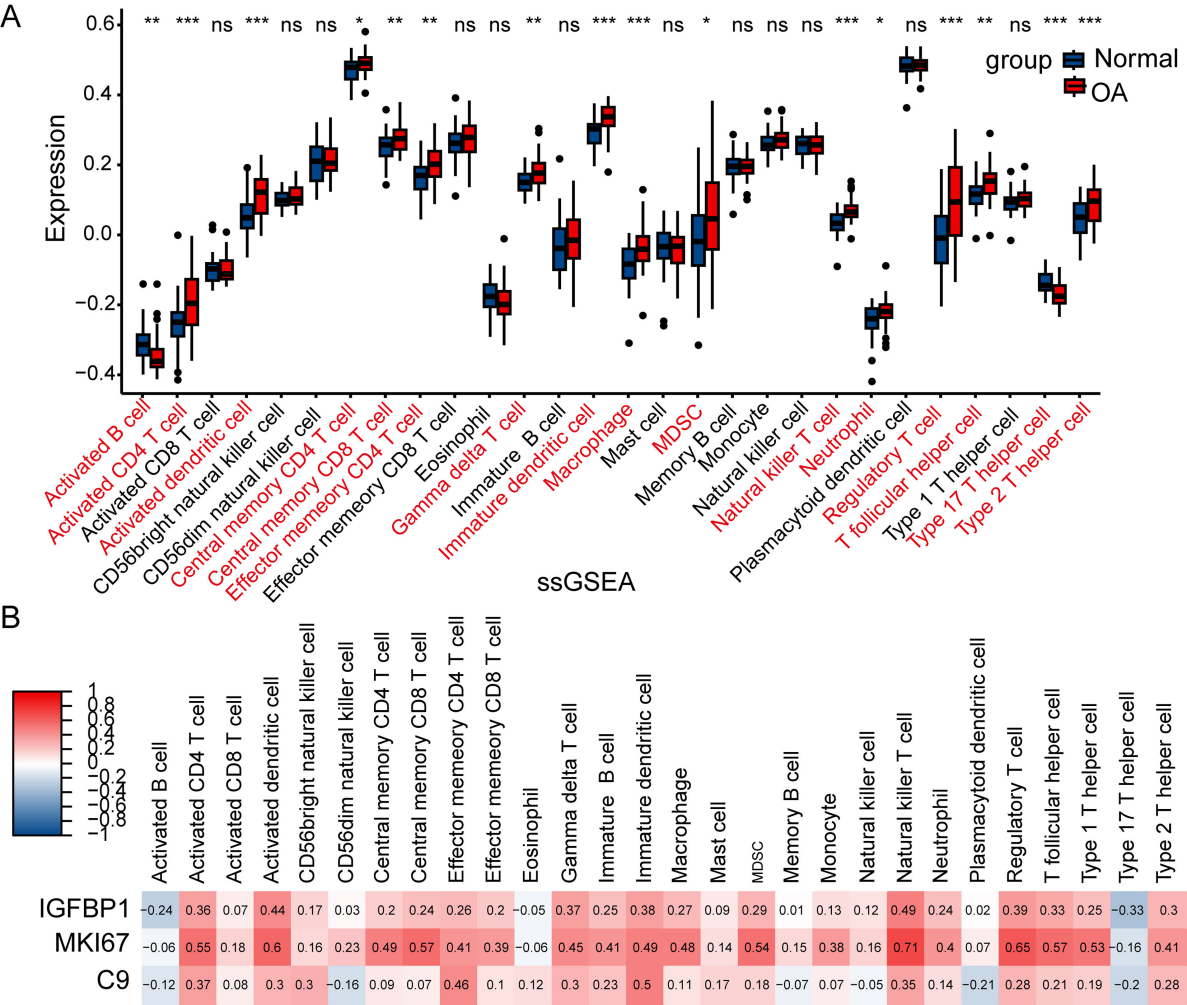
The 28 immune cells and their correlation with the 3 common core genes in OA. **(A)** Heatmap of 28 immune cell expression scores in OA. **(B)** Spearman correlation analysis of the 3 common core genes and 28 immune cells in OA. *p < 0.05; **p < 0.01; ***p < 0.001.

Utilizing the ssGSEA algorithm, we identified a significant increase in the infiltration levels of Activated CD4 T cells, Activated dendritic cells, Central memory CD4 T cells, Central memory CD8 T cells, Effector memory CD4 T cells, Gamma delta T cells, Immature dendritic cells, Macrophages, Myeloid-derived suppressor cells, Natural killer T cells, Neutrophils, Regulatory T cells, T follicular helper cells, and Type 2 T helper cells in OA samples. Conversely, we observed a significant reduction in the infiltration of Activated B cells and T follicular helper cells in these samples (**Figure 8A**). Spearman correlation analysis revealed significant associations between hub genes and immune infiltration levels (**Figure 8B**). IGFBP1 showed a strong positive correlation with Regulatory T cells (ρ = 0.39, *p* < 0.001), Natural killer T cells (ρ = 0.49, *p* < 0.001), and Activated dendritic cells (ρ = 0.44, *p* < 0.01), while negatively correlating with Activated B cells (ρ = -0.24, *p* < 0.05) and Type 17 T helper cells (ρ = -0.33, *p* < 0.05). MKI67 exhibited the strongest positive correlation with Natural killer T cells (ρ = 0.16, *p* < 0.001) and significant correlations with Regulatory T cells (ρ = 0.65, *p* < 0.01) and Effector memory CD4 T cells (ρ = 0.41, *p* < 0.01). C9 demonstrated positive correlations with Immature dendritic cells (ρ = 0.50, *p* < 0.001), Effector memory CD4 T cells (ρ = 0.46, *p* < 0.001), and Activated dendritic cells (ρ = 0.30, *p* < 0.01).

### 3.9 Molecular docking

**Figure 9:**
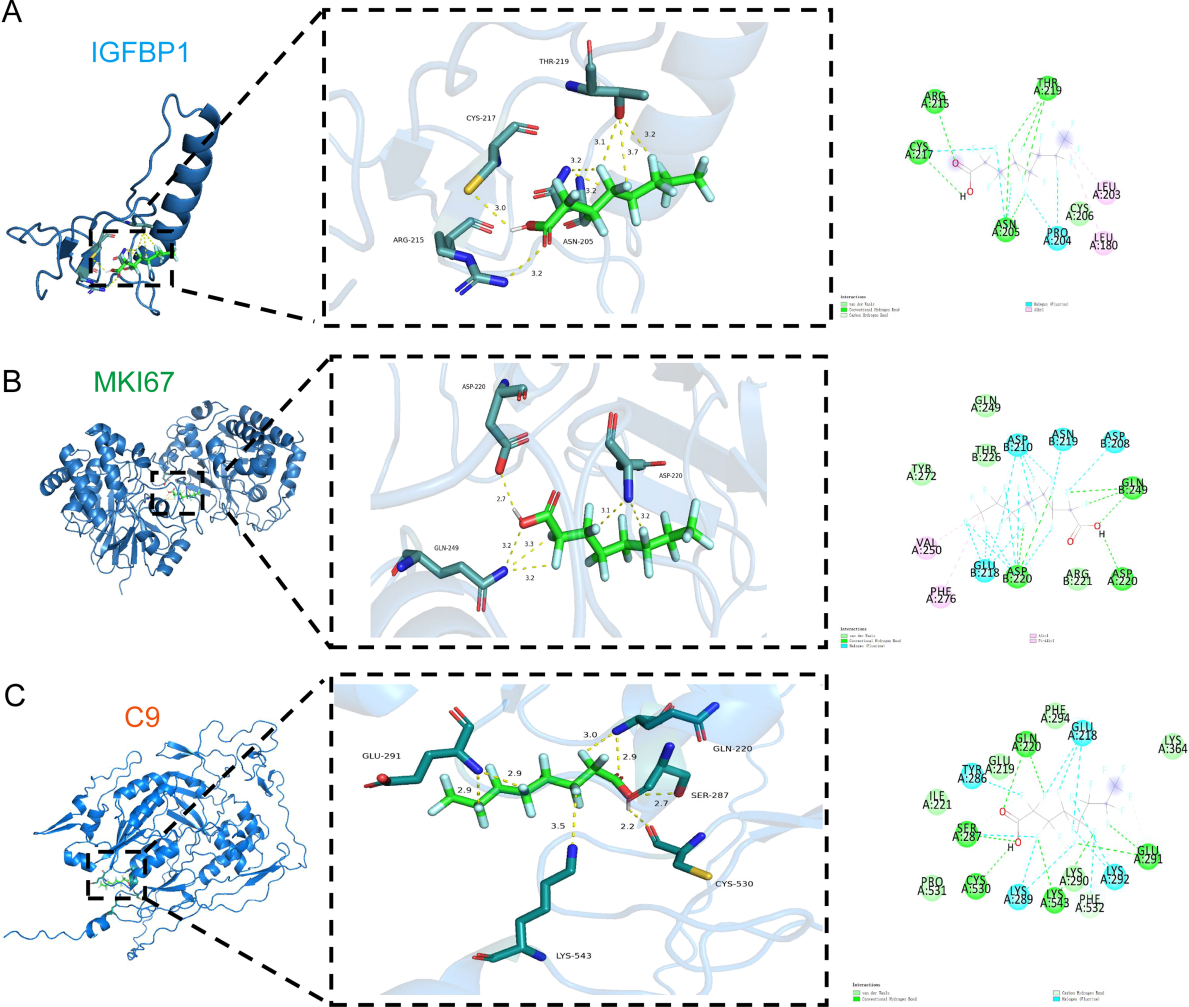
Molecular docking results of PFOA interaction with IGFBP1, MKI67 and C9. **(A)** Molecular docking conformation of PFOA interaction withIGFBP1. **(B)** Molecular docking conformation of PFOA interaction with MKI67. **(C)** Molecular docking conformation of PFOA interaction with C9.

In this study, we selected three target proteins—IGFBP1, MKI67, and C9—for molecular docking analysis. The docking score between PFOA and IGFBP1 was determined to be -6.0 kcal/mol (**Figure 9A**). The carboxyl end of PFOA forms multiple hydrogen bonds with Arg215, Asn205, Thr219, and Cys217 of IGFBP1, with bond lengths ranging from 3.0 to 3.7 Å. This is crucial for the stability of the complex. The perfluoroalkyl chain of PFOA forms specific fluorine-containing bonds with Pro204 and Cys206, and forms van der Waals interactions with hydrophobic residues such as Leu180 and Leu203, further enhancing the binding affinity. The docking score for the interaction between PFOA and MKI67 was -8.5 kcal/mol (**Figure 9B**). The carboxyl end of PFOA forms multiple hydrogen bonds with the key polar residues of MKI67. The hydrogen bond lengths between PFOA and ASP220 (bifunctional site), GLN249 range from 2.7 to 3.3 Å, providing the main anchoring force for the complex. The perfluoroalkyl chain of PFOA forms fluorine atom-mediated halogen bond interactions with residues such as ASP210, ASN219, ASP208, GLU218, and ARG221; meanwhile, residues such as TYR272, THR226, VAL250, and PHE276 participate in the hydrophobic embedding of the perfluoro chain through van der Waals forces, further stabilizing the binding conformation. The docking score between PFOA and C9 was determined to be -7.5 kcal/mol (**Figure 9C**). The carboxyl end of PFOA forms hydrogen bonds with Glu291, Ser287, Gln220, and Lys543 (bond lengths ranging from 2.2 to 3.5 Å). The perfluorinated chain forms halogen bonds with Glu218, Glu219, Lys289, Lys290, and Lys292, while residues such as Ile221, Pro531, Cys530, Phe294, and Phe532 participate in hydrophobic embedding, enhancing the binding affinity.

## 4 Discussion

China is a significant producer and the largest consumer of per- and PFAS (30). Among these, PFOA is the most prevalent and has been demonstrated to exert toxic effects on multiple human physiological systems. PFOA is extensively utilized in the manufacturing of various industrial and consumer products, attributed to its superior hydrophobic and oleophobic properties. Notably, it is employed in industries such as non-stick cookware, cosmetics, and upholstery (31). Nevertheless, its pervasive application in consumer goods, coupled with its prolonged biological half-life, renders it resistant to degradation within the human body (32). PFOA is commonly detected in blood samples from individuals residing in numerous industrialized nations (33). In the United States, the median serum concentration of PFOA typically ranges from 4 to 5 ng/mL, although levels exceeding 20 ng/mL have occasionally been reported. Notably, no significant sex-based differences in PFOA concentrations have been identified (34). Furthermore, PFOA was detected in up to 86% of breast milk samples from lactating women (35). This finding indicates that environmental persistent pollutants can infiltrate ostensibly uncontaminated food supply channels. This has raised significant concerns regarding public health safety, prompting all sectors of society to reassess and rigorously regulate potential sources of pollution, including everyday consumer products, food packaging, and industrial waste.

PFOA exposure has been associated with tumor development in rodent models and with certain human cancers (36). Furthermore, toxicological studies in animals have demonstrated that prolonged dietary administration of PFOA can lead to the induction of liver tumors in rats (37, 38). These observations suggest that PFOA can activate proliferation-related pathways, which may also contribute to synovial hyperplasia in OA. There is an absence of clear biomarkers or therapeutic mechanisms in this context. To date, only large-scale cross-sectional studies have identified a significant positive linear association between PFOA exposure and the incidence of OA (14, 39).

OA, a prevalent chronic condition, significantly impacts the quality of life for hundreds of millions globally. Its etiology is multifaceted, encompassing genetic predispositions, aging, obesity, and environmental factors. To expedite the precise diagnosis and treatment of osteoarthritis, researchers have endeavored to identify clinically valuable biomarkers across various biological dimensions and to pinpoint potential therapeutic targets. For instance, Xia et al. utilized bioinformatics analysis to identify seven ferroptosis-related genes potentially implicated in osteoarthritis synovitis (40). Nonetheless, the diagnostic efficacy of key genes as biomarkers within the cohort remains inadequately explored, with a lack of supplementary experimental data to substantiate these findings. Li et al. identified UGCG and ESYT1 as pivotal genes implicated in lipid metabolism in OA through the application of three machine learning algorithms (41). Additionally, utilizing WGCNA and two machine learning algorithms, Li et al. systematically identified eight immune-related genes associated with osteoarthritis (42). However, the comprehensive diagnostic performance of these genes as indicators of the disease has not been thoroughly assessed, and experimental validation is absent.

To elucidate the potential association between PFOA and OA, we employed network toxicology and bioinformatics integrated with machine learning to identify core genes and molecular mechanisms linking PFOA-related pathways to OA. This was further corroborated through *in vitro* experiments.

In this study, we initially conducted an analysis of the OA transcriptome dataset, identifying 371 differentially expressed genes associated with the condition. These genes exhibited significant enrichment in pathways related to nuclear division, chromosome segregation, rheumatoid arthritis, cytokine-cytokine receptor interaction, and osteoclast differentiation. Furthermore, we extracted 379 genes related to PFOA from the CTD. By using a Venn diagram, we identified a total of 15 DEPFOAs. Enrichment analysis indicated that 15 genes from OA and normal tissues were involved in the regulation of neuroinflammatory response, the p53 signaling pathway, the IL-17 signaling pathway, lipid metabolism and atherosclerosis, and the TNF signaling pathway, among others. These findings align with previous studies (43–46). Subsequently, we identified three key genes—IGFBP1, MKI67, and C9—as potential links between PFOA-related molecular signatures and OA, using six machine learning techniques and WGCNA. Our study demonstrates that these three genes, IGFBP1, MKI67, and C9, possess significant diagnostic potential for predicting OA. In OA cases, the expression levels of these genes are markedly elevated, demonstrating upregulated expression. Clinical samples were collected to validate the expression of the three genes, and the findings corroborated the above results. GSEA revealed that the three key genes associated with osteoarthritis are significantly involved in the regulation of immune infiltration, inflammation, and metabolism. This is consistent with the functional enrichment of DEPFOAs in OA. The study revealed that these three genes are significantly associated with both PFOA-related pathways and OA, suggesting a potential molecular bridge. This research contributes novel insights into the molecular biological underpinnings of OA and underscores the promising potential of personalized medicine approaches in the early detection, precise intervention, and development of tailored therapies for the disease.

Research has demonstrated that the infiltration of immune cells is a significant characteristic in the development and progression of OA (47). Comparative analysis of healthy synovial tissue and that of OA patients indicates a markedly higher presence of T cells within the synovium of the latter group (48). T cells constitute approximately 20% to 25% of the total inflammatory cell population, following macrophages in prevalence (49). These observations underscore the critical role of T cells in the synovial inflammation associated with OA. Furthermore, the augmented infiltration of B cells, plasma cells, natural killer cells, and macrophages plays a significant role in the development and progression of OA (50). In this study, single-sample SSGSEA was employed to evaluate immune cell infiltration in OA, revealing significant differences in the presence of activated CD4 T cells, activated dendritic cells, central memory CD8 T cells, gamma delta T cells, macrophages, natural killer T cells, neutrophils, and T follicular helper cells. Zhang et al. demonstrated that the induction of macrophage polarization towards the M1 phenotype may exacerbate the progression of OA. This process encompasses the secretion of Rspo2 by macrophages, subsequently activating the β-catenin signaling pathway in chondrocytes, thereby facilitating disease progression (51). Furthermore, another study demonstrated that CD4+ T cells exhibited increased polarization towards Th1 cells and elevated cytokine secretion in knee synovial fluid in end-stage OA compared to peripheral blood (52). This localized inflammatory response may exacerbate disease progression and contribute to the persistence of the pathological process. Within the immune microenvironment of OA, cell-associated factors are pivotal, exemplified by the upregulation of IGFBP1, MKI67, and C9 gene expression. These genes are intricately linked to immune cell infiltration and significantly contribute to the pathogenesis of OA.

Insulin-like growth factor-binding protein 1 (IGFBP1) is a secreted protein recognized for its pivotal role in modulating the bioactivity of insulin-like growth factors (IGFs), thereby influencing a wide array of biological processes across different life stages, including development, adulthood, and senescence. These processes encompass proliferative dynamics, cellular migration, aging mechanisms, autophagy, vascular development, differentiation, and apoptotic pathways (53, 54). Moreover, IGFs and their binding proteins are integral to the development, maintenance, and repair of the skeletal system (55). These findings underscore the significance of these molecules in OA pathogenesis. Recent research has identified IGFBP1 and IGFBP3 as pivotal contributors to the pathological mechanisms underlying OA. These proteins are implicated not only in the extensive degradation of the extracellular matrix but also in promoting cell death and fostering an inflammatory milieu, collectively precipitating the characteristic response pattern observed in OA (56). This groundbreaking discovery offers a novel paradigm for the evaluation and management of OA. External research has identified IGFBP1 as a pivotal component in the hepatic-osteoendocrine axis, with the capacity to facilitate osteoclastogenesis and bone resorption. Experimental data demonstrated a significant elevation in the bone resorption marker C-terminal peptide fragment following the administration of recombinant IGFBP1 in murine models, suggesting an enhancement of bone resorption activity by IGFBP1 (57). Furthermore, the inhibition of osteoclast activity effectively mitigated the bone resorption process, thereby impeding the progression of OA (58). The present findings suggest that targeting the inhibition of IGFBP1 function may constitute a viable strategy for decelerating or preventing the progression of OA. By suppressing osteoclast activity and bone resorption, it is possible to mitigate the degradation of articular cartilage, thereby preserving joint function. Empirical studies have demonstrated that both experimental animals and humans exposed to PFOA frequently exhibit disruptions in the endocrine system, with the upregulation of IGFBP1 potentially serving as one of the response mechanisms. This upregulation may subsequently influence glucose and lipid metabolism, potentially contributing to related metabolic disorders (59). An increasing body of research indicates that OA is characterized by metabolic disorders (60). These findings offer novel insights suggesting that environmental factors, such as exposure to PFOA, may be associated with the onset of metabolic diseases. Furthermore, IGFBP1 may serve as a crucial molecule connecting environmental exposure to metabolic disorders.

MKI67, commonly referred to as Ki67, is a nuclear protein that plays a critical role in chromosome segregation and the transcription of ribosomal RNA during mitosis (61). The primary function of Ki67 is to facilitate the dispersal and independent movement of chromosomes, rendering it a significant marker of cellular proliferation. Given that tumor cells typically demonstrate abnormally high proliferative activity, Ki67 has emerged as a pivotal biomarker in numerous oncological studies. For example, the Ki67 index is widely used as a prognostic marker in cancer (62). Elevated Ki67 expression is typically indicative of a tumor’s highly aggressive biological behavior (63). Furthermore, it has been observed that increased Ki67 expression facilitates the enhancement of energy metabolic pathways, such as glycolysis, oxidative phosphorylation, and glutamine metabolism, which supply the requisite energy and substrates for ribosomal component synthesis (64). It is well-established that chondrocytes, along with other cellular entities implicated in the pathophysiology of OA, such as synoviocytes and immune cells, require a sufficient energy supply to support their proliferation and functional activities. Elucidating the interactions at this level during the processes of inflammation and repair in OA could yield insights for the development of novel therapeutic strategies. Empirical evidence indicates that exposure to PFOA enhances the expression of genes associated with cellular proliferation, such as MKI67. For instance, in cultured neonatal mouse ovaries, PFOA at concentrations of 50 μM and 100 μM resulted in a 5.8-fold and 6.3-fold increase in Ki67 transcript levels, respectively (65). his observation implies that PFOA exposure not only enhances cell proliferation but may also interfere with normal cell cycle regulation. Similarly, combined exposure to PFOA and other environmental chemicals has been shown to elevate Ki67 expression in stem cells (66). Suggesting that these chemicals may work together to promote cellular overproliferation.

C9 is a critical element of the complement system, contributing to the formation of the membrane attack complex, which is essential in the host’s defense against bacterial and other pathogenic invasions. As a single-chain glycoprotein, C9 is classified within the membrane attack complex/perforin-like superfamily (67, 68). Research indicates that the activation of the complement system is markedly elevated in instances of acute arthritis, particularly when induced by bacterial infections. In this context, C9 is pivotal in initiating the inflammatory response, acting as a crucial component within the complement cascade. For instance, in cases of acute bacterial arthritis, there is a substantial accumulation of C9 within synovial tissue, which plays a role in cytolysis and inflammatory processes (69). Conversely, in chronic degenerative conditions such as osteoarthritis (OA) without acute inflammation, the deposition of C9 is not significant. This observation implies that the activation of the complement system is predominantly linked to acute inflammatory responses rather than chronic degenerative lesions (69). A separate investigation demonstrated a significant reduction in C9 serum levels in a rat model of OA following treadmill exercise, indicating that moderate physical activity may alleviate inflammation and pain in OA patients (70). Collectively, these studies elucidate the intricate involvement of C9 and the complement system in the pathophysiological mechanisms of arthritis. Furthermore, they suggest that exercise could serve as an effective non-pharmacological intervention to enhance symptom management and improve the quality of life for individuals with arthritis. PFOA has the potential to disrupt lipid metabolism and cellular functions through its impact on the expression of peroxisome proliferator-activated receptors alpha and gamma (PPARα and PPARγ). In a similar vein, the expression of the C9 gene is intricately associated with inflammatory responses and immune function. Interplay between PFOA and C9 expression may exacerbate chronic inflammation-related conditions (71).

Integrating the known toxicological properties of PFOA with the functional implications of the three hub genes, we propose several potential mechanistic links between PFOA exposure and OA pathogenesis. First, PFOA is known to induce oxidative stress and activate transcription factors such as NF-κB, which may upregulate MKI67 expression, thereby promoting abnormal synovial fibroblast proliferation and contributing to joint degradation. Second, PFOA-induced metabolic disturbances, particularly in lipid and glucose metabolism via PPARα/γ pathways, may alter IGFBP1 expression, potentially disrupting chondrocyte metabolism and bone remodeling balance. Third, PFOA exposure may activate the complement system, with C9 serving as a key effector molecule, thereby enhancing local immune responses and promoting a chronic inflammatory microenvironment conducive to OA progression. Although these mechanism hypotheses are derived based on integrated bioinformatics analysis and known toxicological pathways, they need to be further validated through functional experiments.

Molecular docking analyses have elucidated the binding sites and interaction patterns of PFOA with target proteins, demonstrating that all targets exhibit favorable docking affinities with PFOA. The interactions primarily involve direct effects on key genes through the formation of hydrogen bonds with amino acid residues such as ARG, CYS, and GLU. Nonetheless, a comprehensive series of *in vitro* and *in vivo* experiments is required to substantiate the accuracy and physiological relevance of these predictions conclusively.

The robustness of our diagnostic model was further supported by external validation in GSE51588 (AUC = 0.939), indicating good generalizability beyond the discovery dataset. Simultaneously, our study is subject to several limitations. Initially, the dataset utilized at the commencement of the study comprised a relatively limited number of samples. This necessitates validation using a larger sample pool to mitigate bias and enhance the robustness and generalizability of the findings. Secondly, while initial *in vitro* experiments validated the expression of the three key genes in synovial tissue samples, further *in vivo* experimental methodologies, such as employing knockout or overexpression mouse models, are necessary to comprehensively investigate the functions of these genes and their intrinsic associations with the OA process under *in vivo* conditions. Notably, the current bioinformatics and network toxicology framework identifies correlations rather than direct causal relationships. Specifically, because the OA transcriptomic datasets do not contain individual−level PFOA exposure information, we cannot infer that PFOA exposure directly causes the observed changes in IGFBP1, MKI67, and C9 expression in OA patients. The observed associations merely suggest that these genes are simultaneously linked to PFOA (based on prior toxicological evidence) and to OA (based on differential expression analysis). Whether PFOA directly regulates these genes or indirectly influences their expression through broader immune and metabolic remodeling, and whether such regulation contributes causally to OA pathogenesis, remain to be determined through prospective cohort studies or experimental models. Thirdly, the data sources underpinning this study primarily originate from secondary analyses of publicly accessible databases, which may still harbor some bias despite thorough screening and statistical processing. Finally, within the constraints of the existing analytical framework, it is possible that certain equally significant signature genes were overlooked. These under-explored factors may play pivotal roles in the pathogenesis of OA. Fourth, the qRT−PCR validation was performed on a limited number of synovial tissue samples (6 OA patients and 6 controls). Although the expression trends were consistent with the bioinformatics analysis and reached statistical significance, the small sample size may affect the generalizability of these findings. Larger independent cohorts are needed to confirm the diagnostic value of IGFBP1, MKI67, and C9.

## 5 Conclusion

This research highlights a significant molecular association between PFOA−related genes and OA, suggesting that PFOA exposure may be linked to OA pathogenesis at the transcriptomic level. However, due to the lack of exposure history in the public datasets, causal inference cannot be made from the current analysis. Utilizing various machine learning algorithms, we identified three genes—IGFBP1, MKI67, and C9—as markers associated with OA in contexts of PFOA exposure. Subsequent analysis using ROC curves, alongside rigorous *in vitro* experiments, validated their high efficacy in the diagnosis of OA. Our findings reveal a significant association between these key genes and immune cell interactions, suggesting that PFOA exposure may be linked to OA pathology through potential modulation of the immune response. This discovery not only broadens the understanding of OA pathogenesis but also serves as a valuable reference for interdisciplinary research on environmental toxins and human health.

## Data Availability

The original contributions presented in the study are included in the article/**Supplementary Material**. Further inquiries can be directed to the corresponding author.

## Supplementary material

Supplementary Table 1 DEGs identification.

Supplementary Table 2 Sheet 1 The significant GO entries enriched by the potential target genes. Sheet 2 The significant KEGG pathway by the potential target genes.

Supplementary Figure 1 The heatmap exhibiting the correlations between DEPFOAs.

Supplementary Figure 2 ROC curve of the nomogram in the independent external validation set GSE51588 (10 normal, 40 OA). AUC = 0.939 (95% CI: 0.846–1.000).
