# Supplementary figures and images for: From algorithm to verification: based on network toxicology and machine learning, the immunomodulatory role of IGFBP1/MKI67/C9 in perfluorooctanoic acid-induced osteoarthritis was discovered, and a diagnostic model was constructed
Source: Front Immunol. 2026 May 28;17:1700638. doi: 10.3389/fimmu.2026.1700638 (PMC13253507; doi:10.3389/fimmu.2026.1700638)

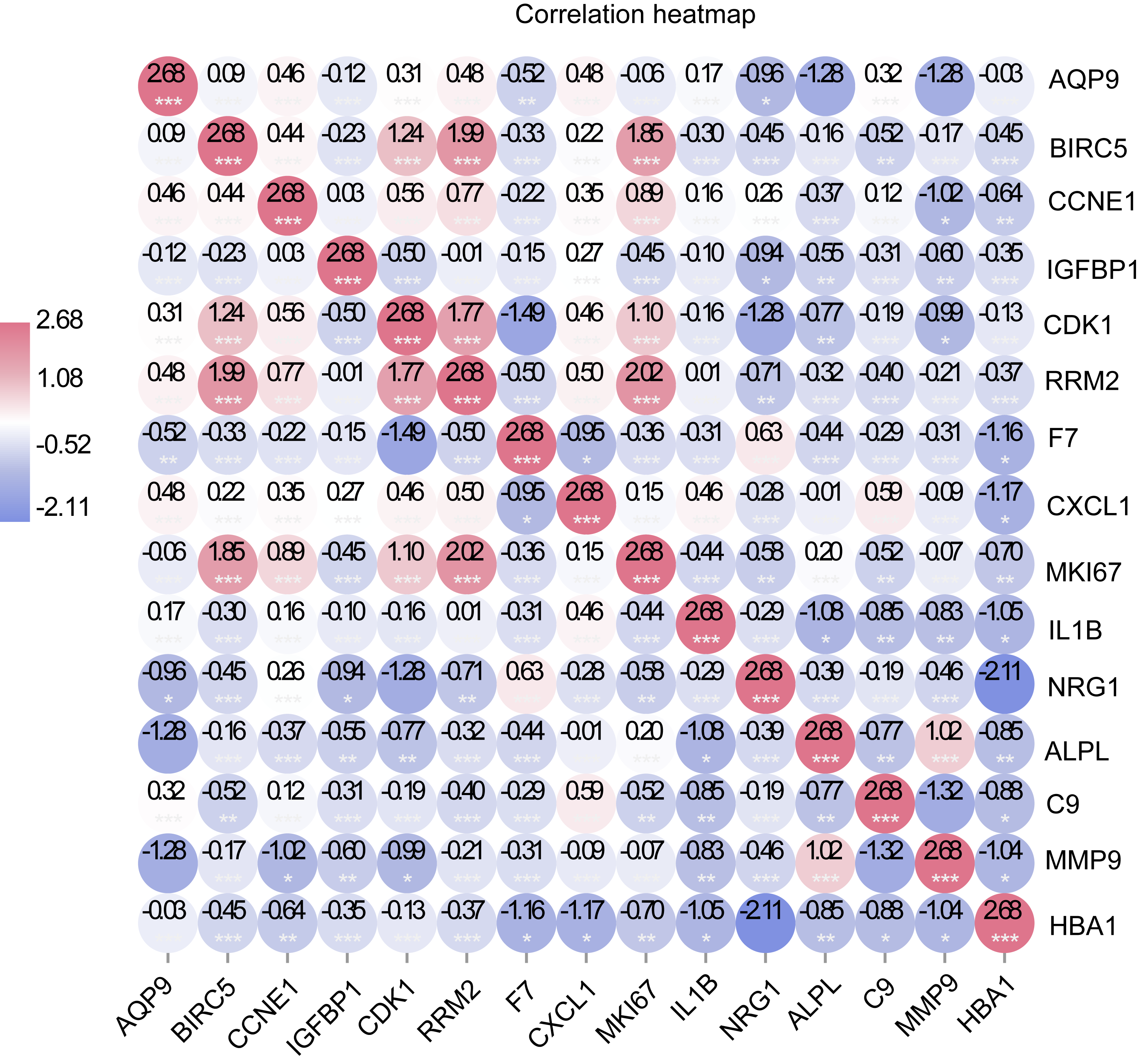

Supplement: Supplementary Figure 1 — The heatmap exhibiting the correlations between DEPFOAs. [file Image1.tif]

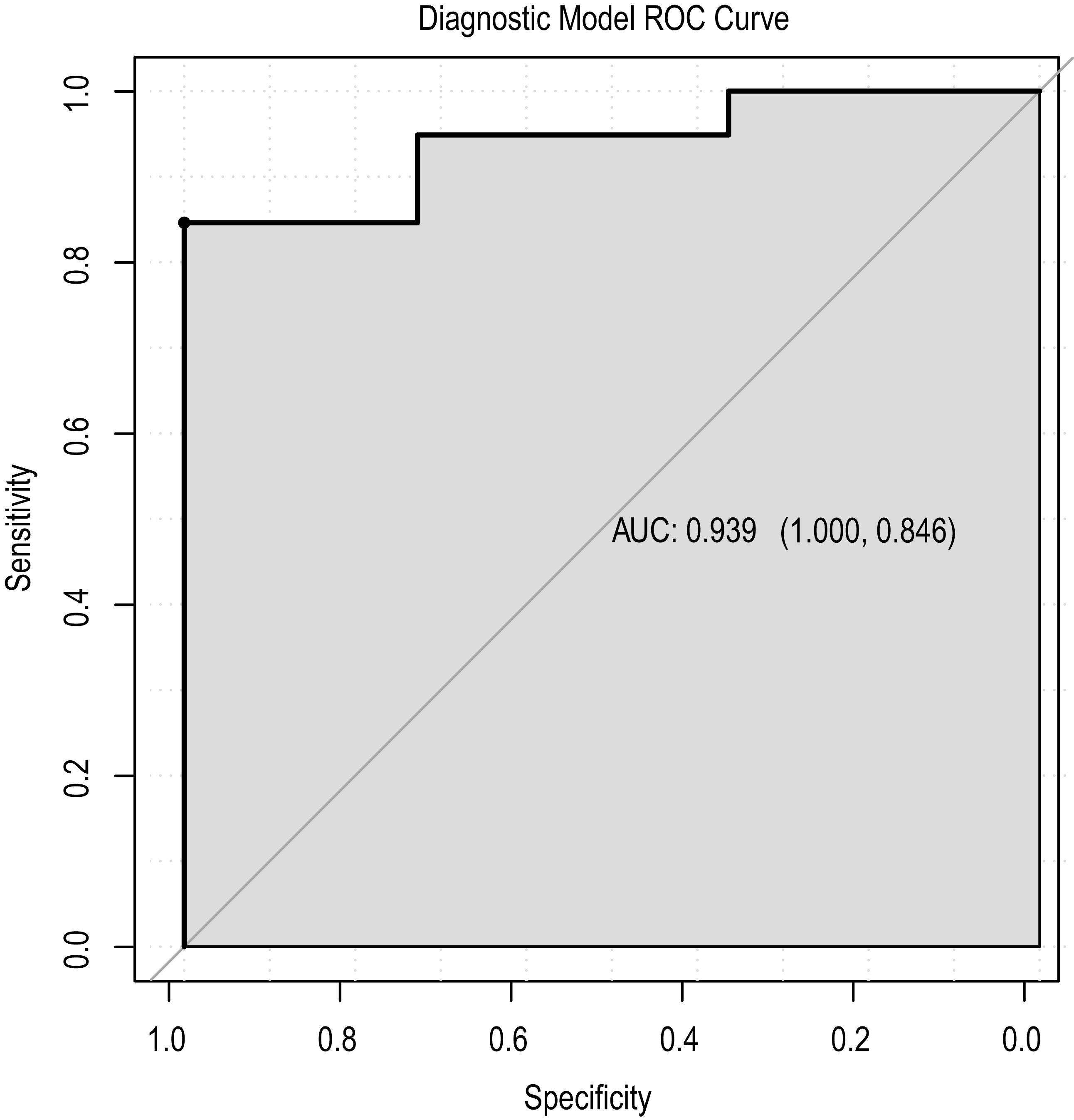

Supplement: Supplementary Figure 2 — ROC curve of the nomogram in the independent external validation set GSE51588 (10 normal, 40 OA). AUC = 0.939 (95% CI: 0.846–1.000). [file Image2.tif]
